# An image and video dataset of nesting green sea turtles with annotated data

**DOI:** 10.1038/s41597-024-04336-3

**Published:** 2024-12-27

**Authors:** Irwandi Hipiny, Hamimah Ujir, Ruhana Hassan, Abang Arabi Abang Aimran

**Affiliations:** 1 Universiti Malaysia Sarawak, Faculty of Computer Science and Information Technology, Sarawak, 94300 Malaysia; 2 Universiti Malaysia Sarawak, Faculty of Resource Science Technology, Sarawak, 94300 Malaysia; 3 Sarawak Forestry Corporation, Sarawak, 93250 Malaysia

**Keywords:** Zoology, Research data

## Abstract

Photo- and video-based reidentification of green sea turtles using their natural markers is far less invasive than artificial tagging. An RGB camera mounted on a man-portable rig, was used to collect video data on Greater Talang Island (1 °54’45″N 109 °46’33″E) from September to October 2022, and September 2023. This islet is located 30 minutes offshore from the Sematan district in Southwest Sarawak, Malaysia. In total, 42 videos of 42 unique individuals were captured, from which 14,471 video frames were extracted and annotated with a bounding box each. Through manual inspection, the annotation data were diligently checked and edited if necessary. Additionally, 130 selected frames were further annotated each with a region-of-interest mask containing only the carapace. These data have the potential to aid researchers in training computer vision models for various tasks such as counting, segmentation and individual reidentification.

## Background & Summary

The iconic green sea turtles (*Chelonia mydas*) are an important part of Sarawak’s coastal ecosystem. They are recognized as endangered under the International Union for Conservation of Nature (IUCN) Red List of Threatened Species^1^, and are totally protected in Sarawak, under the Wild Life Protection Ordinance 1998^2^. To aid in their conservation effort, several active nesting sites in Sarawak were declared as a Totally Protected Area (TPA)^3^ to limit interactions with humans and support population growth via the establishment of natural hatcheries. To estimate the population size and understand migratory behavior, nesting individuals captured within the TPAs are tagged by affixing a metal tag with a unique ID to each front flipper, enabling future reidentification. Records of turtle landings and nests at Greater Talang and the surrounding areas have been maintained since 1946^4^.

Unfortunately, the artificial tagging practice places significant stress on the sea turtles^5^. Moreover, there is a high risk of tag loss^6^. While reidentifying individuals based on photos of their natural markers (i.e., facial scales, flipper scales, scutes pattern, and pigmentation pattern), holds promise, its efficacy is hampered by the time required to process the data. Hence, automating the tasks using computer models has emerged as an ideal solution. These natural markers are either found on the facial areas, flippers, or carapace. The carapace, being the largest body part of a green sea turtle, is easier to photograph in a less invasive manner. Examples of natural markers found on the carapace include the scutes pattern^7^ and the pigmentation pattern (on the posterior part)^8^.

**Fig. 1:**
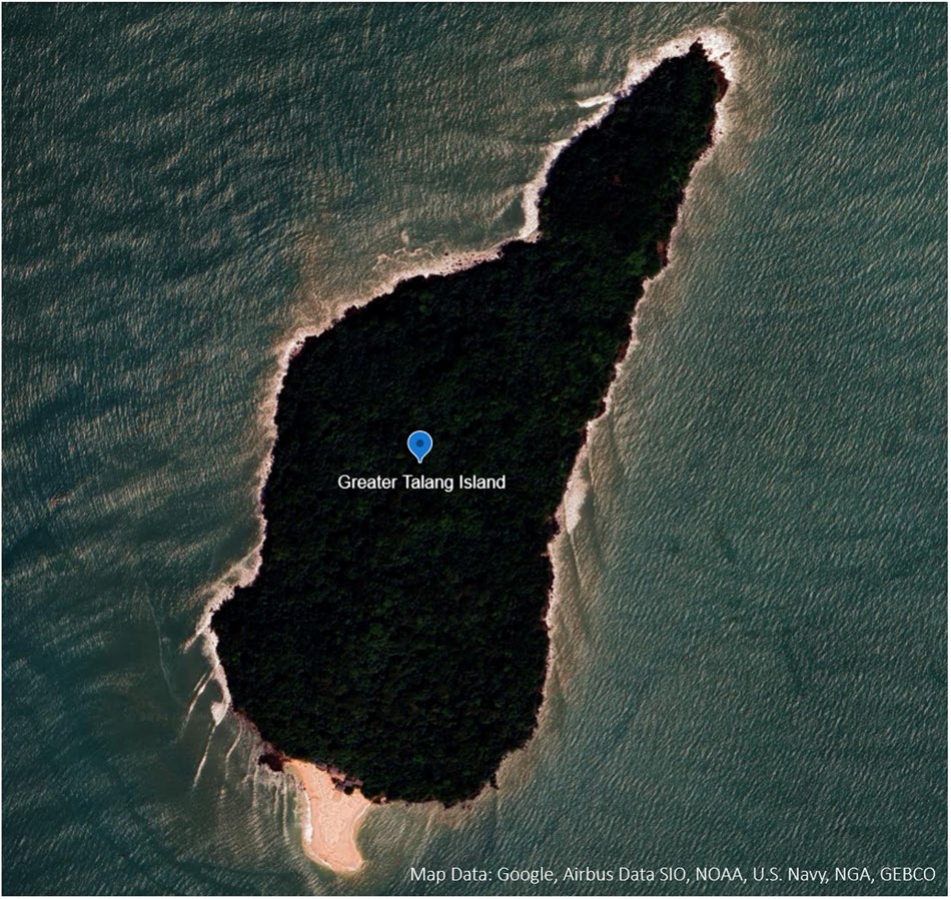
A satellite image of Greater Talang Island, Sarawak, Malaysia, sourced from Google Earth^24^. The sandy beach area is situated at the southern tip of the island, where most of the nesting activities are concentrated.

This motivated the collection of video data at Greater Talang, one of the four islets that form Talang-Satang National Park. The islets are surrounded by shallow coral reefs that provide shelter and resting grounds for grazing sea turtles. In 2017, Greater Talang recorded the highest number of landings, with 1,881, followed by Lesser Talang with 838 landings, and Greater Satang with 215 landings^9^. Landings at Greater Talang peak during the dry season^10^, i.e., from May until September each year, and subside during the rough monsoon weather from November until April of the following year. During the monsoon season, the rough sea disperses the sand at the small beach area at the southern tip of Greater Talang, refer to Fig. 1, rendering it unsuitable for egg laying^11^.

Videos were collected from September to October 2022 and September 2023 using an RGB camera mounted on a man-portable rig. The rig, including the camera, weighs approximately 8.5 kg and can be carried on a person or temporarily planted on a porous surface. An ideal setup would consist of many stationary cameras arranged in a grid to ensure maximum coverage of the beach area. However, due to cost limitations and permit’s constraints, the decision was made to employ a man-portable rig instead.

There is a common consensus that green sea turtles randomly select their nest excavation site^12^. Another theory suggests beach topography as the determining factor^13^. Therefore, permanently planting the camera rig at an identified hotspot is possible. Nevertheless, a mobile setup increases the number of possible recordings since the data collection team can quickly relocate to another individual once the current recording is completed.

**Fig. 2:**
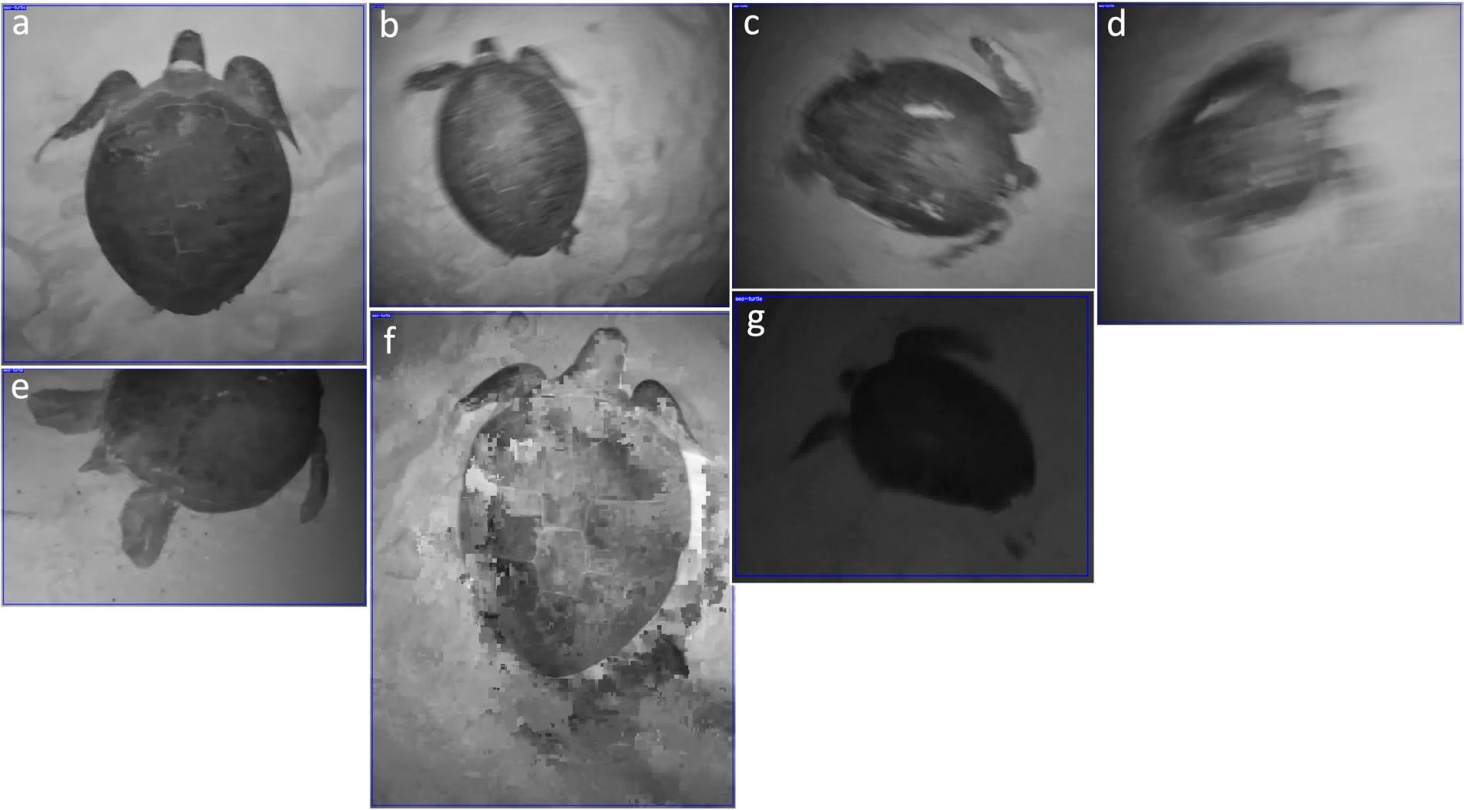
Bounding box image samples with varying quality levels: (**a**) good, (**b**) slight blurring, (**c**) strong blurring, (**d**) excessive blurring, (**e**) partially-visible (occlusion), (**f**) JPEG compression, and (**g**) illumination change. Image blurring and out-of-plane transformations were due to the camera’s motion invoked by the rig operator’s movement and/or strong winds. Illumination varies due to the amount of moonlight received. The dimensions of the bounding box are nonuniform and depend on the size of the enclosed sea turtle. A short video showcasing the bounding box annotations across continuous frames is available on the author’s YouTube page (https://youtu.be/aLRbj9gdFEg).

**Fig. 3:**
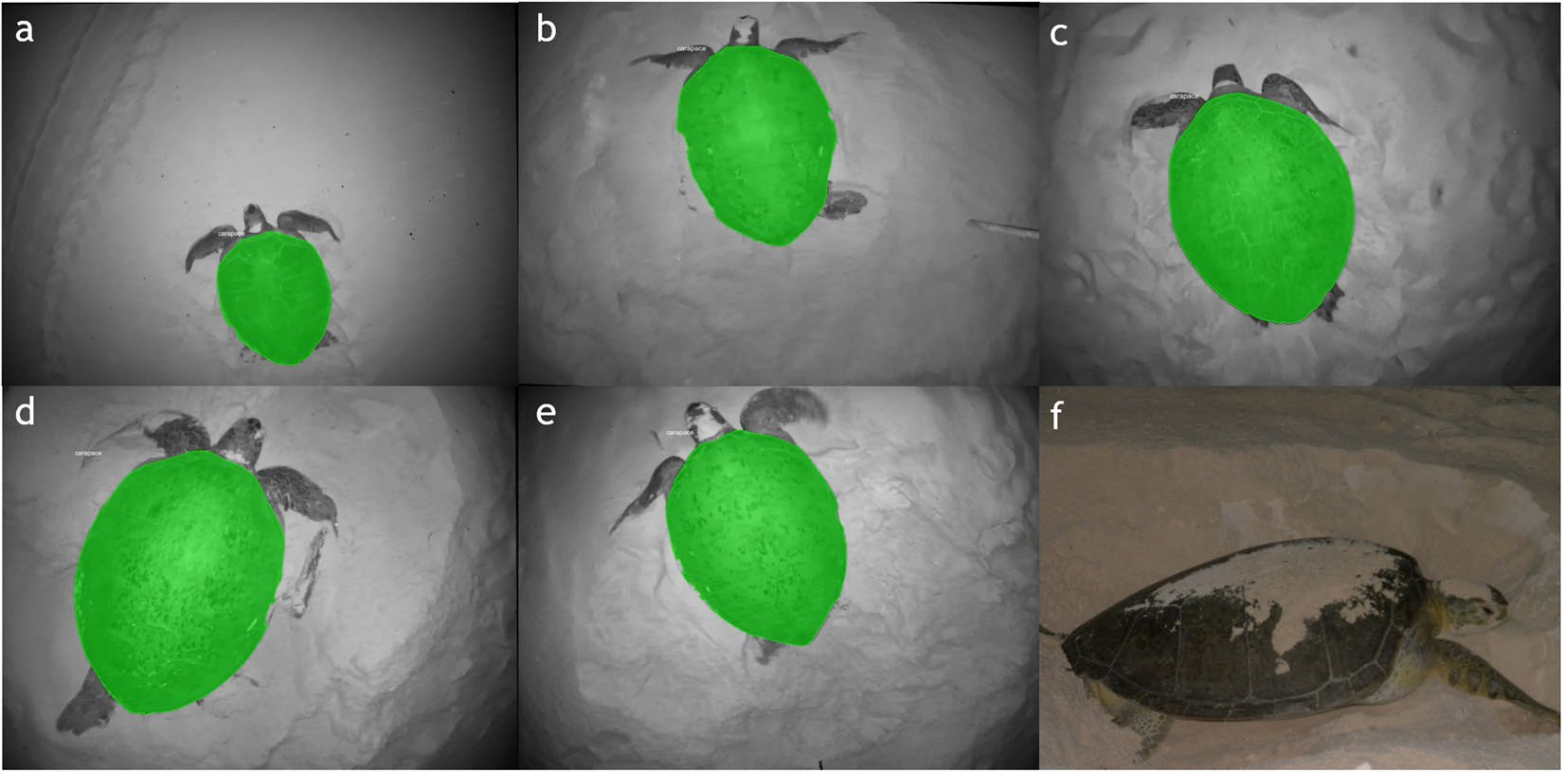
(**a**) - (**e**) Visualized ROI mask samples. In most cases, the edges of the carapace are easily identifiable, except for the top and bottom parts where extra attention is required to visually separate the hard shell from the soft body parts, i.e., the head, body, and flippers. A side-view image^25^, (**f**), of a green sea turtle is provided as a visual reference.

A video frame with a bounding box annotation indicates the presence of a green sea turtle, and vice versa. Samples with varying quality levels are shown in Fig. 2. An ROI mask separates the foreground, specifically, the carapace, from the background. Annotation data for each frame are stored inside a paired JSON file. The visualized ROI mask samples are shown in Fig. 3. The research team had published two prior works using the annotated dataset^14^. The first article^15^ reported the performance of a pre-trained YOLOv7 model^16^ on a bounding box detection task. The second article^17^ evaluated the instance segmentation performance of Mask R-CNN^18^ by comparing it to manually annotated ROI masks on contrast-equalized images.

Public wildlife video and/or photo datasets are abundant, but only a small number exist for sea turtles. SeaTurtleID2022^19^ is a large-scale, long-span dataset containing timestamped photos of free-swimming loggerheads, captured during snorkeling surveys from distances ranging from 7 meters to a few centimeters. The dataset contains 8,729 photos of 438 individuals collected over a 13-year period. Annotation data include identity, encounter timestamps, and segmentation masks for body parts (i.e., head, carapace, and flippers). The photos were captured at large time intervals, thus enabling the use of time-aware splits during training and validation. Another large public dataset, TurtleSpot Taiwan^20^, contains 3,515 crowdsourced sea turtle sighting photos of 762 individuals, captured underwater or on land. The included species are Greens, Hawksbills, and Olive Ridleys. The annotation data included sighting location, date, time, depth, observation method, and photographs of the whole body as well as the left and right faces. Smaller public datasets, such as ZindiTurtleRecall^21^ and PANDANCHELOMY^7^, are also available, but these datasets typically have minimal annotation data. The two datasets contain photos of sea turtles captured on land in a controlled environment. Side-by-side comparisons of the datasets are shown in Table 1.

**Table 1:** Comparison with existing datasets.

	SeaTurtleID 2022^19^	TurtleSpot Taiwan^20^	ZindiTurtle Recall^21^	PANDAN CHELOMY^7^	GTST-2023^14^
Species	Loggerhead.	Green, Hawksbill and Olive Ridley.	Green and Hawksbill.	Green (Juvenile).	Green.
Modality	Photos.	Photos.	Photos.	Photos.	Photos and videos.
Environment	Underwater.	Both.	On land.	On land.	On land.
Dataset size	8,729 photos of 438 individuals.	3,515 photos of 762 individuals.	300 photos of 100 individuals (training set).	70 photos of 16 individuals.	42 videos of 42 individuals. 14,471 photos.
Target body part(s)	Whole body.	Whole body, left and right face.	Top, left, and right face.	Carapace.	Carapace.
Annotation data	Identity, encounter timestamp, and segmentation masks.	Crowd-sourced: Sighting location, date, time, depth and observation method. Inferred: Identity, sex, life stage, behaviour, taxa and physical abnormality (if any).	Identity and face pose.	Identity.	Identity, bounding box coordinates and labels, and segmentation masks.

## Methods

**Fig. 4:**
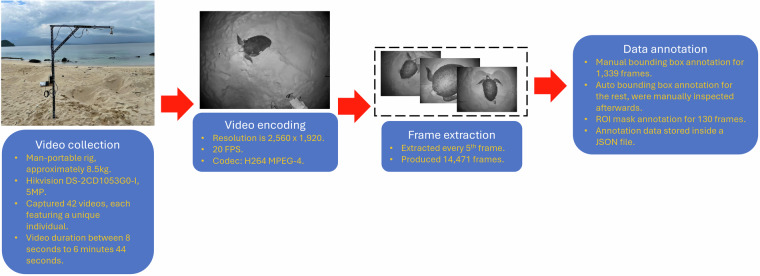
The multistage dataset production process.

The production of the dataset^14^ involved a multistage process, shown in Fig. 4. This section describes each stage in detail.

The experimental protocol described in this section was reviewed and approved by a research review committee under the jurisdiction of Sarawak Forestry Corporation. The permits (permit numbers: SFC.810-4/6/1 (2021) - 73 and SFC.810-4/6/1 (2023) - 075) were issued only after receiving a successful approval.

**Fig. 5:**
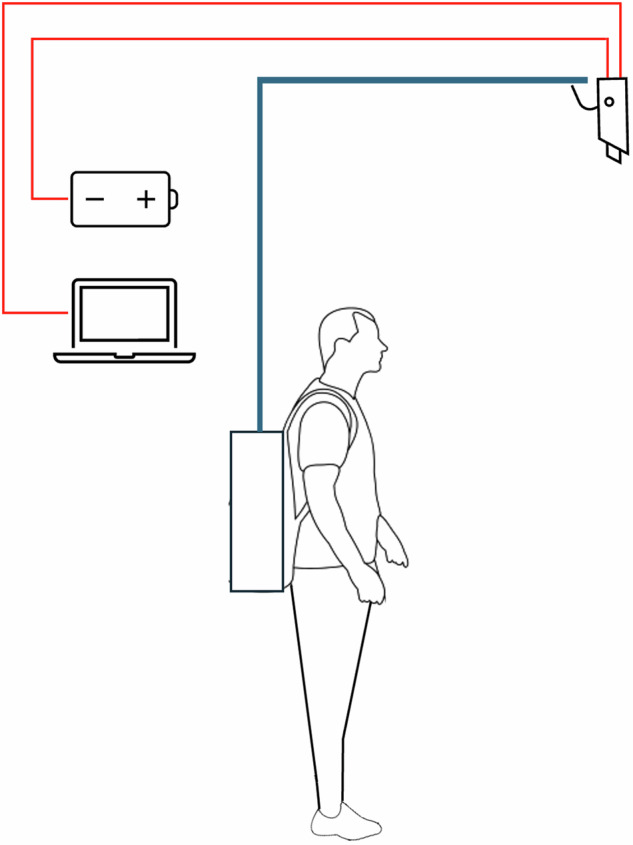
The man-portable camera rig.

Stage one involves video collection, which is performed during two separate periods: September to October 2022 and September 2023. A man-portable rig, as shown in Fig. 5, weighing approximately 8.5 kg was used to capture the videos. The rig has a CCTV camera, powered by an external battery with a capacity of 50,000 milliamp-hours, ensuring extended operational time. The rig was built using extendable poles, adjustable to a height of 1.5 to 2.1 meters, and can be hoisted up to 3 meters above the ground.

The recordings were made at night, in near darkness, with only moonlight as the primary source of illumination. The camera does emit infrared light, but it is minimal. In a typical scenario, park wardens alert the data collection team to the presence of a nesting green sea turtle on the beach. The team begins to approach and record only after the individual has finished covering the new nest with sand. In some cases, recording starts only after the park wardens have completed the tagging and measurement activities, when the green sea turtle is already making its way back to the sea. During recording, the team positions the downwards-facing camera at an appropriate height and as perpendicular as possible to the carapace. The camera position is maintained for a period of time to obtain sharp and in-focus images of the carapace.

Stage two is video encoding. A total of 42 videos, each featuring a unique green sea turtle, were successfully collected over the two data collection periods. The videos were encoded using the H264 MPEG-4 codec, at an original resolution of 2,560 x 1,920 and a frame rate of 20 FPS. The total size for all videos is 1.66 GB.

During stage three, video frames were extracted every fifth frame. This is a good interval for capturing sea turtles’ laboured movement on land, resulting in 14,471 video frames. The videos were not trimmed, thereby including background noise inside the data.

Stage four involves the iterative and exhaustive process of adding annotation data to each video frame to provide context. The annotation data can later be used to train or validate machine learning models. There are two annotation types: bounding boxes and ROI masks. Bounding box annotations were manually added to 1,339 video frames extracted from all 42 videos. An augmented version of the set was used to train a Roboflow Object Detection (Fast) model^22^ (mAP 93.0%, precision 97.6%, and recall 87.0%) to produce bounding box annotations for the remaining frames. The model’s confidence threshold and overlap threshold were both set at 50.0% during inference. These threshold values were relaxed to ensure that a large number of bounding box images would be collected, with false positives removed later during manual inspection. The complete list of preprocessing steps and augmentations is shown in Table 2. Next, the bounding box annotations were inspected and edited if necessary. The first annotator performed an initial pass to detect and flag anomalies. A two-person team then performed a quality check pass, making edits if necessary. Manual checks are essential since missed or erroneous detection is still possible with the trained object detector model. A similar setup was used to produce the ROI masks. The frame selection criteria were as follows: the target (i.e., the carapace) must be in focus, experience minimal blurring, and be substantially large. The annotator used Labelme software^23^, a graphical image annotation tool, to carefully mark the carapace’s outline for 130 selected video frames. A two-person team then visually checked the created masks and edited any discrepancies.

**Table 2:** Preprocessing steps and augmentations for the training data.

Preprocessing steps	Auto-orient
	Rescaled to 640 × 480
	Tile: 2 rows x 2 columns
Augmentations	Flip: Horizontal and Vertical
	Rotation between − 15. 0 ° and + 15. 0 °
	Blur: up to 2.5px
	Noise: up to 0.1% of pixels

## Data Records

The complete dataset is archived on Kaggle under the title ‘GTST-2023’^14^. The dataset contains 42 videos and two image sets: Set A, which includes 14,471 video frames with bounding box annotations, and Set B, which includes 130 frames with ROI mask annotations. All images had a resolution of 2,560 x 1,920 pixels and were stored in RGB format. For Set A, each image is paired with a JSON file containing the following key-value pairs:

- predictions: This key corresponds to the properties of the predicted bounding box.
  - x: The horizontal center point of the detected object.
  - y: The vertical center point of the detected object.
  - width: The width of the bounding box.
  - height: The height of the bounding box.
  - confidence: The prediction’s confidence score.
  - class: The deep learning object detector predicts a single class, i.e., ‘sea-turtle’.
  - detection_id: Identifier for Roboflow’s hosted inference call.
  - image_path: Path to the image.
  - prediction_type: Prediction type used by Roboflow.
- image: This key corresponds to the image.
  - width: Width of the image.
  - height: Height of the image.

For Set B, each image is paired with a JSON file containing the following key-value pairs:

- version: This key corresponds to the dataset’s version.
- flags: This key corresponds to flags, if any.
- shapes: This key corresponds to shape object(s). Only one shape object (i.e., carapace) per image.
  - label: The identifier for the shape object.
  - points: An array of piecewise points that mark the shape object’s outline.
  - group_id: The group identifier.
  - shape_type: Shape’s type is set as ‘polygon’.
  - flags: Flags, if any.
- imagePath: This key corresponds to the path of the image.
- imageData: This key corresponds to the image’s data in binary form.
- imageHeight: This key corresponds to the height of the image.
- imageWidth: This key corresponds to the width of the image.

The entire dataset^14^ is approximately 3.66 GB in size.

## Technical Validation

Data annotations were evaluated based on established criteria to ensure consistency, especially in ambiguous cases. For the autogenerated bounding box annotations, annotators consistently applied the definition for Class ‘sea turtle,’ i.e., the bounding box was retained (or added) if the enclosed object could be visually identified as a sea turtle, determined by consensus. Bounding box annotations for blurred and poorly illuminated images were retained to ensure that the source data were broad and substantial enough to be representative of real-world conditions. Bounding box images containing an individual with partially occluded body part(s) were also retained. As described in the Methods section, the bounding box annotations underwent a two-pass check. During the initial pass, the annotator performed a check to detect and flag anomalies. In the second pass, a two-person team conducted a quality check, making edits where necessary. Ambiguous cases were resolved through majority voting, involving the two-person team and the annotator. The same process was applied when producing the ROI mask annotations.

Video frames in the dataset^14^ exhibit common image deformations such as blurring, in-plane and out-of-plane transformations, illumination changes, and scale changes. Additionally, the videos were purposely not trimmed to only contain good frames of the carapace. This ensures the substantial presence of background noise, which is very useful for training and validating robust computer models.

## Usage Notes

The bounding boxes and ROI masks can be visualized using any suitable image viewer that supports the JSON annotation format, such as the LabelMe software^23^.

## Data Availability

No additional code is supplied together this manuscript.
